# Werner syndrome through the lens of tissue and tumour genomics

**DOI:** 10.1038/srep32038

**Published:** 2016-08-25

**Authors:** Mari Tokita, Scott R. Kennedy, Rosa Ana Risques, Stephen G. Chun, Colin Pritchard, Junko Oshima, Yan Liu, Peter K. Bryant-Greenwood, Piri Welcsh, Raymond J. Monnat

**Affiliations:** 1 Department of Medicine Division of Medical Genetics, University of Washington, Seattle, WA USA; 2 Department of Pathology, University of Washington, Seattle, WA USA; 3 Department of Radiation Oncology, MD Anderson Cancer Center, Houston, TX USA; 4 Department of Laboratory Medicine, University of Washington, Seattle, WA USA; 5 Department of Medicine, Chiba University, Chiba, Japan; 6 Department of Pathology, John Burns School of Medicine, University of Hawaii at Manoa, Honolulu, HI USA; 7 Department of Genome Sciences, University of Washington, Seattle, WA USA

## Abstract

Werner syndrome (WS) is the canonical adult human progeroid (‘premature aging’) syndrome. Patients with this autosomal recessive Mendelian disorder display constitutional genomic instability and an elevated risk of important age-associated diseases including cancer. Remarkably few analyses of WS patient tissue and tumors have been performed to provide insight into WS disease pathogenesis or the high risk of neoplasia. We used autopsy tissue from four mutation-typed WS patients to characterize pathologic and genomic features of WS, and to determine genomic features of three neoplasms arising in two of these patients. The results of these analyses provide new information on WS pathology and genomics; provide a first genomic characterization of neoplasms arising in WS; and provide new histopathologic and genomic data to test several popular models of WS disease pathogenesis.

Werner syndrome (WS, OMIM #277700) is the prototypic human autosomal recessive adult progeroid (or ‘premature aging’) syndrome. WS patients develop features reminiscent of premature aging beginning in the second decade of life. These include bilateral cataracts, graying and loss of hair, scleroderma-like skin changes, diabetes mellitus and osteoporosis, and are accompanied by an elevated risk of clinically important, age-associated diseases. Cancer and atherosclerotic cardiovascular disease are the most common causes of death, at a median age of 54 years^1^,^2^,^3^,^4^,^5^.

A wide spectrum of different tumor types has been reported in WS patients, with 2/3 of these represented by six tumor types: thyroid epithelial neoplasms, malignant melanoma, meningioma, soft tissue sarcomas, leukemia and pre-leukemic conditions of the bone marrow, and primary bone neoplasms. The elevated risk of these neoplasms ranges from 53.5-fold for melanoma versus population controls, to 8.9-fold for thyroid epithelial neoplasms^6^. Despite the large number of reports of neoplasia and multiple neoplasms in WS patients, remarkably few reports have documented any genomic feature of neoplasia beyond confirming the presence of *WRN* disease-causative mutations (see, e.g.^7^,^8^,^9^).

Our aim in work reported here was to delineate genomic, molecular and cellular features of WS using tissue from four WS patients coming to autopsy. Two of these patients were Japanese-American sisters first reported in 1966 in a seminal manuscript that delineated WS clinical and pathologic features and the autosomal recessive mode of inheritance^1^. Our results also provide the first comprehensive pathologic and molecular characterization of tissue and tumors from *WRN* mutation-typed WS patients coming to autopsy.

## Results

### Clinical and pathologic findings

We studied four WS patients who came to autopsy after dying at ages ranging from 45 to 57 years. All four patients were *WRN* mutation-typed by the Werner Syndrome International Registry to confirm their clinical diagnoses of WS^3^,^10^ (Tables 1 and 2). Clinical records, pathology specimens and frozen tissue were identified and used to characterize clinical, gross and histopathologic features of WS in these patients, and for molecular, histopathologic and genomic characterization including a first genomic characterization of three neoplasms arising in two of our patients.

We used previously published WS pathology reports^1^,^9^,^11^,^12^,^13^,^14^,^15^,^16^,^17^,^18^,^19^,^20^,^21^ to develop a list of recurrent clinical and histopathologic findings in WS, then scored these findings in each of our patients (Tables 2 and 3, Supplementary Methods and Results). Scleroderma-like skin changes were present, though variable, in all four patients. These changes included epidermal atrophy, hyper-keratosis, dermal fibrosis and subcutaneous fat atrophy, and were most clearly delineated in the two oldest of our patients (Patients 1 and 2; see below).

Cardiovascular findings were present in all four patients, included systemic atherosclerotic vascular disease involving the aorta, coronary and cerebral arteries in order of descending severity (all four patients); old and recent septal myocardial infarction (Patient 2); and intimal and medial fibrosis of medium-sized peripheral arteries (Patient 1). Gross pathologic evidence of cerebrovascular disease included a remote left cerebral hemispheric infarct with associated midbrain, pontine and cerebellar atrophy (Patient 3), together with two potential additional infarcts in Patient 1 (soft lesions in the left caudate and cingulate gyrus noted at the time of brain cutting, though not sampled for histopathology). Two patients had above-the-knee amputations of one leg (Patient 1) or both legs (Patient 2) to treat intractable foot ulcers.

**Figure 1:**
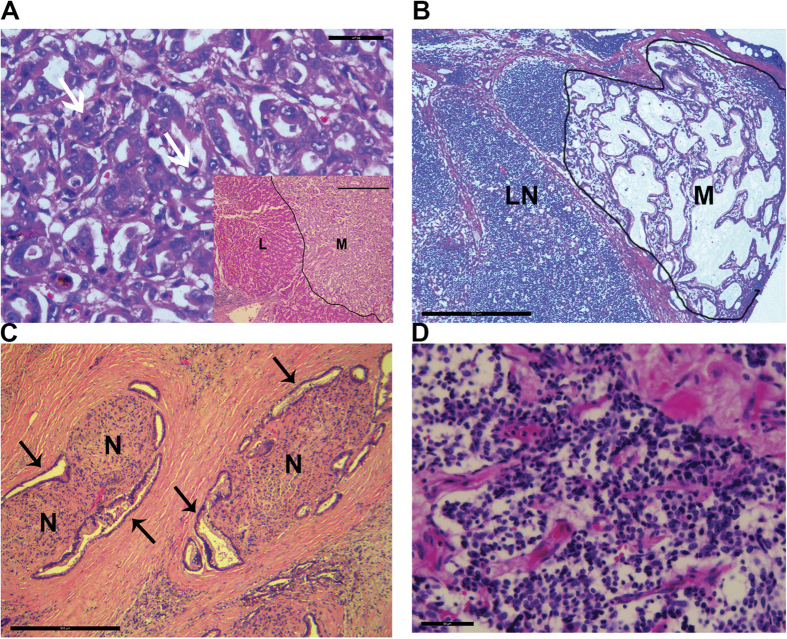
Histopathological features of neoplasms in Werner patients. (**A**) H&E staining of pancreatic adenocarcinoma liver metastasis in Patient 3. Arrows indicate mitotic figures. Scale bar, 50 μm. Inset shows liver (L) and adjacent metastasis (M) separated by a sharp boundary (dark line). Inset scale bar, 500 μm. (**B**) H&E staining of pancreatic adenocarcinoma lymph node metastasis in Patient 4 growing in and expanding the subcapsular sinus of an abdominal lymph node (LN). Scale bar, 500 μm. (**C**) H&E staining of pancreatic adenocarcinoma perineural invasion in Patient 4. Arrows mark invading carcinoma. N, nerve. Scale bar, 500 μm. (**D**) H&E staining of a pulmonary carcinoid in Patient 4. Scale bar, 100 μm.

Neoplastic disease was present in three patients at autopsy. Metastatic pancreatic adenocarcinomas with liver and lung metastases were found in Patients 3 and 4 with associated chronic pancreatitis in both patients, and in Patient 4 with biliary obstruction and liver necrosis. Figure 1 shows the histopathologic appearance of a pancreatic adenocarcinoma liver metastasis with readily detectable mitotic activity in Patient 3 (Fig. 1A), and of subcapsular sinus lymph node metastasis and abdominal perineural invasion in Patient 4 (Fig. 1B,C). An additional 0.7cm carcinoid tumor was found in the left upper lung in Patient 4 at autopsy, and was examined by histopathology and molecular profiling (Fig. 1D). Multiple small meningiomas were present in Patient 2, though were not sampled for histopathology at the time of autopsy^1^.

Acute lung pathology contributed to the death of all four patients: bronchopneumonia affecting one or both lungs in Patients 1–3, together with extensive pancreatic adenocarcinoma lung metastases with lung parenchymal destruction, hemorrhage and inflammation in Patients 3 and 4. Two patients had thyroid nodules: one with focal calcification and necrosis (Patient 2), and the other with focal fibrosis and nodule formation (Patient 4). A more detailed list of gross and histopathologic findings at autopsy for all four WS patients is given in Supplementary Results.

### Molecular characterization of neoplasms

In order to characterize genomic features of the neoplasms identified in Patients 3 and 4 we used targeted capture and next generation sequencing of 234 genes related to cancer diagnosis, treatment and prognosis on the UW Oncoplex platform version 4.0; http://tests.labmed. washington.edu/UWOncoPlex^22^). The UW-OncoPlex platform can reliably detect single nucleotide (SNP/SNV) variation in samples with ≥10% tumor DNA content, together with small insertions and deletions (indels), gene amplification and a small subset of gene fusions^22^. This sensitivity of detection allowed us to detect somatic mutations in our three neoplasms that had estimated tumor-derived fractions of 20% (pancreatic adenocarcinoma metastases from Patients 3 and 4) and 50% (pulmonary carcinoid in Patient 4).

We identified non-synonymous somatic SNVs in *KRAS* and *TP53* in both pancreatic adenocarcinomas, together with a stopgain SNV in *SMAD4* in Patient 4. In Patient 4 there was also potential LOH (Table 4). Many genomic regions of both pancreatic carcinomas also displayed evidence of potential copy number variation. In contrast to the confident calling of SNP/SNV mutations, these would require additional work to confirm and characterize.

Recent whole genome sequencing of pancreatic adenocarcinoma has suggested several subtypes defined on the basis of the extent and nature of genomic rearrangements^23^. We thus determined whether either pancreatic carcinoma sample had mutations in 47 genes known to influence genomic stability that are included in the Oncoplex version 4.0 panel^24^. However, none of these 47 genes, including *BRCA1*, *BRCA2* and *PALB2,* displayed known deleterious, disease-associated mutations (Table S2). Genomic capture and sequencing of DNA isolated from the incidental pulmonary carcinoid identified in Patient 4 did not identify somatic coding SNP or indel variants, but did reveal potential CNVs that again will require additional work to confirm and characterize.

### Telomere length and mtDNA mutations in WS tissue

We assayed telomere length and using highly accurate DNA extracted from frozen liver tissue from WS Patients 1 and 2 that was taken at the time of autopsy^1^, and highly accurate Duplex Sequencing to characterize mtDNA mutation frequency and spectrum in the same DNA samples. Relative telomere length was measured by qPCR as previously described^25^,^26^, and compared with DNA from normal liver samples from two control donors. Relative telomere lengths of liver DNA from WS Patient 1 (age 57) and Patient 2 (age 51) were 0.82 and 0.83, respectively. These relative lengths were slightly longer than the two normal liver controls that had relative telomere lengths of 0.68 (Control donor 1, age 69) and 0.70 (Control donor 2, age 78). These results are consistent with the expected telomere shortening observed with older age, and argue against a substantial, global shortening of telomeres in WS despite differences in the age and gender of our two patients and controls.

Both mitochondrial dysfunction and mtDNA alterations have been postulated to contribute to WS disease pathogenesis (reviewed in ref. 27). Moreover, a mouse model of WS with a deletion of the *Wrn* helicase domain has an increased frequency of mtDNA point mutations in liver and myocardial tissue^28^. Evidence for increased oxidative stress has been reported for WS patients as well as WS mouse models^27^,^29^, and WRN has been shown to play a role in the repair of oxidative DNA damage^30^. A key prediction from these observations is that the frequency of G > T/C > A mutations—a hallmark of oxidative DNA damage^31^—should be increased in the mtDNA of WS patients.

**Figure 2:**
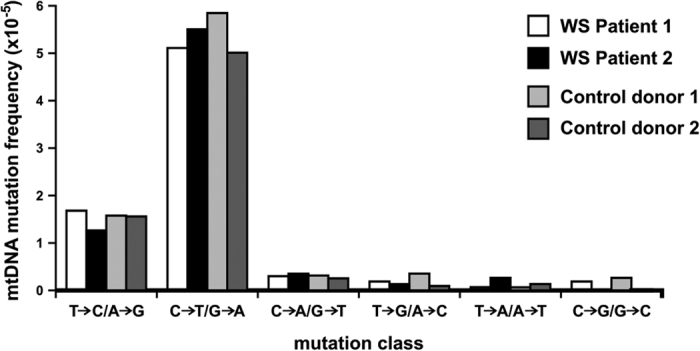
Frequency and spectrum of specific base substitution mutations in mtDNA from WS patient and control liver. Frequency and spectrum of specific base substitution mutations as determined by Duplex Sequencing in mtDNA isolated from liver tissue of two WS patients (WS patients 1 and 2) and two control donors (Control donors 1 and 2).

In order to test this prediction we used highly accurate Duplex Sequencing methodology^32^,^33^ to quantify the frequency and determine the molecular spectrum of mtDNA mutations in WS patient tissue samples. We observed no significant difference in the global mtDNA mutation frequency in WS patients as compared with controls: WS Patient 1: 3.72 × 10^−5^ and WS Patient 2: 3.32 × 10^−5^, versus Control liver 1: 4.27 × 10^−5^; and Control liver 2: 3.5 × 10^−5^. We did not identify differences in base substitution mutations by molecular type, and note the comparative lack of G > T/C > A mutations (Fig. 2). These findings are consistent with our previous analyses of mtDNA base substitution frequencies and spectrum in other control individuals, and suggest that extensive mtDNA mutagenesis resulting from oxidative DNA damage is not a prominent driver of WS disease pathogenesis^31^.

### Characterization of senescent cells in WS tissue

**Figure 3:**
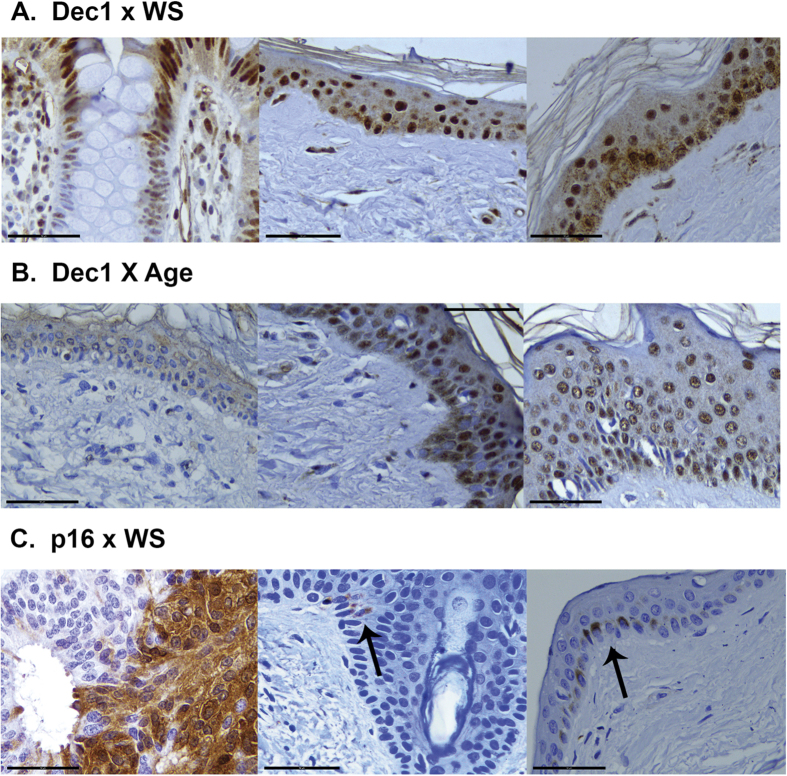
Immune staining detects senescent cell markers in Werner Syndrome patient tissue. (**A**) DEC1 x WS. Left panel: DEC1 immunostaining in an ulcerative colitis tissue sample (positive control). Note gradient of positive staining (basal/bottom to apical/top) typically observed in colonic crypts. Middle and right panels show DEC1 immunostaining in autopsied abdominal wall skin from WS patient 1 (middle panel) and WS patient 2 (right panel). (**B**) DEC1 x Age. DEC1 immunostaining of control skin from 4 month old control donor (left panel), a 49 year old control donor (middle panel) and a 50 year old control donor (right panel).(**C**) p16 x WS. Panels show irregular p16 immunostaining typically observed in high grade ovarian serous carcinomas (positive control, left panel); in abdominal wall skin from WS patient 1 (middle panel); and in abdominal wall skin from WS patient 2 (right panel). Arrows in middle and right panels indicate small clusters of p16-immunostained cells. All images are 40 X with scale bars representing 50 μm.

Cellular senescence, a hallmark of WS^34^,^35^, was analyzed in tissue samples from WS Patients 1 and 2 by immunostaining tissue sections to detect DEC1 and p16, two cellular markers of senescence^36^,^37^. DEC1 staining was readily detectable and robust in control as well as WS patient skin samples (Fig. 3A,B). DEC1 staining was both epidermal as well as dermal, with dermal staining detected as scattered dermal fibroblasts, endothelial staining in small dermal vessels and skin appendage staining. WS Patient 1 and Patient 2 showed increased numbers of DEC1-stained cells together with increased intensity of DEC1 staining when compared to age-matched controls (Fig. 3A). There was very little detectable DEC1 staining in TMA skin from a 4 month old control donor, in contrast to skin from 49 yo and 50 yo control donors (Fig. 3B).

Immunostaining for p16 was performed using the mouse monoclonal primary antibody (clone E6H4) and staining kit (CINtec Histology kit, MTM Laboratories) used previously to quantify senescent cells in human skin during aging^36^. We included a high grade serous ovarian carcinoma sample as a positive control, which displayed strong p16 staining (Fig. 3C)^38^,^39^, but found as previously reported^36^ only infrequent p16 stained cells or small cell clusters in control skin samples. These were limited to the epidermis. We did not observe an increased proportion of p16-positive cells beyond these rare stained cells or clusters in skin samples from either of our WS patients (Fig. 3C), and observed the same in control tissue (Supplementary Figure 1).

## Discussion

Werner syndrome has long served as a model of genetically determined human premature aging^1^,^40^. In order to better characterize and understand potential mechanisms driving WS disease pathogenesis, we analyzed clinical, pathologic and molecular features in four WS patients coming to autopsy (Table 1).

The clinical phenotype of WS is well-delineated, though variably penetrant as a function of age^1^,^4^,^40^. The most consistent early consistent changes (‘cardinal features’) of WS have been used to establish clinical diagnostic criteria for WS (see the International Registry of Werner Syndrome: http://www.wernersyndrome.org for additional detail). Our four patients had a ‘definite’ (Patients 1 and 2), ‘probable’ (Patient 3) or ‘possible’ (Patient 4) clinical diagnoses of WS by these criteria prior to the molecular confirmation of *WRN* mutations (Tables 1 and 2).

Gross and histopathologic changes at autopsy in our four patients closely resembled prior autopsy findings in WS^1^,^9^,^11^,^12^,^13^,^14^,^15^,^16^,^17^,^18^,^19^,^20^,^21^ (Table 3), and together document multisystem disease affecting the skin, cardiovascular and respiratory system in all four patients. There was advanced atherosclerotic peripheral vascular disease together with evidence of recent or remote myocardial and cerebral infarction, and three instances of neoplasia: metastatic pancreatic adenocarcinoma with liver and lung metastases in Patients 3 and 4; multiple meningiomas in Patient 2; and a pulmonary carcinoid in Patient 4. Pancreatic adenocarcinoma and carcinoid tumors have been reported in WS patients, though WS patients do not appear to be at increased risk of developing either tumor type in contrast to meningiomas^6^.

Despite WS being a genomic instability/cancer predisposition syndrome^41^,^42^, there has been no detailed molecular or genomic characterization of neoplasms in WS patients (see, *e.g.*^7^,^8^). We were thus particularly interested in characterizing genomic changes in the three neoplasms identified in two of our patients. Targeted gene capture and sequencing was used to identify mutations and CNVs in 234 genes mutated in many human tumors^22^ (Table 4). Somatic SNV and CNV mutations were readily detectable in both pancreatic carcinomas, though the genes and mutations did not differ from sporadic pancreatic adenocarcinomas arising in the general population^23^. We identified potential CNVs in the pulmonary carcinoid from Patient 4, though no other convincing SNVs among our target gene set (Table 4; additional results not shown). Pulmonary carcinoids have a unique mutational signature consisting of loss of function mutations in genes involved in chromatin remodeling and modification, together with infrequent mutation of genes that are recurrently mutated in small cell and large cell neuroendocrine lung tumors (e.g., *TP53* and *RB1*)^43^.

Cellular senescence is a hallmark of both WS and the aging process^34^. Cellular senescence mediated by TP53^44^ or p16-dependent pathways^37^,^45^ can be detected by immunostaining to detect expression of Differentiated Embryo Chondrocyte-expressed gene 1 (DEC1) protein, a TP53-induced transcription factor overexpressed in premalignant, senescent tumors^46^,^47^, or p16 protein. We were able to identify robust DEC1 staining, though only rare p16 immunostained cells or small cell clusters, in skin from WS patients. One explanation for this differential staining pattern is that DEC1 and p16 identify different senescence pathways^37^,^44^,^48^,^49^ that may be variably active in WS. Consistent with this idea is our recent analysis of gene expression in WS patient fibroblasts that identified several mechanistically distinct, senescence-associated gene sets that were significantly enriched for differentially expressed genes in WS patient fibroblasts. These gene sets included DNA damage/telomere stress, oxidative stress, oncogene-induced and senescence-associated secretory phenotype (SASP) senescence pathways^50^,^51^.

WRN plays a well-documented role in telomere maintenance, and telomere dysfunction has been postulated to play a role in WS pathogenesis as well as cellular senescence^52^,^53^,^54^. While it has been reported that skin of WS patients had shorter telomeres than normal controls^55^, we did not observe substantially shorter telomeres in liver DNA from two WS patients compared with two control donors. This assay, however, was limited by the small number of patients and age differences between cases and controls and thus, it would be able to identify only substantial differences between the two groups, if present. While substantial average shortening of telomeres was not found in WS, we cannot rule out the presence of small average differences or differences in the distribution of telomere lengths within an individual. Additional more sensitive assays will be needed to detect critically short telomeres (e.g., by STELA assay), or the consequences of telomere dysfunction regardless of length (e.g., TIF assays).

Mitochondrial dysfunction and mtDNA mutagenesis have also been implicated in the pathogenesis of WS. We used highly accurate Duplex Sequencing to quantify and characterize mtDNA mutations in WS tissue samples^31^,^32^. While we were able to readily detect and quantify mtDNA mutations in WS patients and control donors, neither the frequency or spectrum of mutations differed between patients or two controls or control donors in prior analyses^31^. These results argue against WS disease pathogenesis being driven by oxidative DNA damage-driven mtDNA mutagenesis and mitochondrial dysfunction. We could not, by this approach, exclude the possibility that WS patients accumulate additional mtDNA mutation types—e.g., large deletions—that may increase as a function of donor age^31^,^33^, nor do these results rule out other types of mitochondrial dysfunction in WS.

Our analyses of tissue, tissue sections and tumor DNA from four well-characterized, *WRN* mutation-confirmed WS patients coming to autopsy provide new information on WS cellular, molecular and genomic features. Our results are internally consistent despite the small sample size, and do not provide support for several popular mechanistic hypotheses to explain WS disease pathogenesis: senescent cell accumulation, marked global telomere shortening, or mtDNA mutation accumulation. The most striking finding in all of these analyses was the absence of substantial differences–quantitative or qualitative– between WS tissue or tumor specimens and comparable control samples. One suggestion from these findings is that WS pathogenesis and acquired disease risk reflect may reflect subtle differences in the rate of accumulation, rather than the nature, of tissue, cell, and molecular alterations, together with persistent and simultaneous upregulation of several different senescence- and disease-promoting pathways or processes^50^. Thus WS may indeed be among the most useful genetically-determined models for the study of human aging and age-associated disease, as was first suggested 50 years ago^1^.

## Methods

### Autopsied WS patients

Patient 1, a Japanese American woman with clinically-diagnosed Werner syndrome, died at age 57 of fulminant bronchopneumonia and pulmonary edema. She was previously reported as case M8a/HMc in ref. 1. Patient 2 was the younger affected sister of Patient 1. She died at age 51 from cardiac complications secondary to pulmonary bronchopneumonia, and had been previously reported as case M8c/MI^1^. Patient 3 was a 43-year-old Japanese-American man with molecularly confirmed loss-of-function mutations in the *WRN* gene. He died at age 43 of metastatic pancreatic cancer, and has been previously reported^9^. Patient 4 was a Caucasian woman of English descent who died of metastatic pancreatic adenocarcinoma at age 47. She was identified through the International Registry of Werner Syndrome (http://www.wernersyndrome.org/registry/registry.html), where homozygous loss-of-function mutations in the *WRN* gene were first documented. She was included in our prior analysis of tumor risk and spectrum in WS^6^, though not further reported. Additional clinical detail on all four patients is provided in Supplementary Methods.

Clinical records, pathology specimens (slides and blocks), frozen tissue and laboratory data from these patients were collected and analyzed with informed consent from next of kin of Patients 1–3, and with informed consent from Patient 4 under approval from and in accordance with guidelines established by the University of Washington Human Subjects Internal Review Board (UW#44017) and the Fred Hutchinson Cancer Research Center Internal Review Board (FHCRC protocol #9039). All analyses and methods were carried out in accordance with relevant guidelines established by these Review Boards.

### Additional WS autopsy case-finding

In order to identify previous reports of autopsy findings in WS patients, we used systematic literature searches to identify case reports of WS in English or other languages, then identified among these the subset that included autopsy findings alone, or in conjunction with neoplasia (see Supplementary Methods). Case reports of WS individuals residing in Japan were identified by searching J-EAST (http://sciencelinks.jp/j-east/) and PubMed (http://www.ncbi.nlm.nih.gov/pubmed/). Reports of WS patients with or without neoplasms residing outside Japan were identified by searches in PubMed and Google Scholar (http://scholar.google.com/). A more detailed description of this case-finding strategy has been published, together with a comprehensive list of all reported neoplasms arising in WS patients^6^.

### DNA isolation and sequencing

Tumor DNA, isolated from macro-dissected sections of formalin-fixed, paraffin-embedded (FFPE) tissue, was used for sequencing library preparation as previously described^22^,^56^. The resulting libraries were analyzed by the targeted capture and next-generation sequencing of 234 genes involved in human neoplasia on the UW OncoPlex platform, version 4 (http://tests.labmed.washington.edu/UW-OncoPlex)^22^.

DNA was isolated from frozen liver tissue from WS Patients 1 and 2 taken at the time of autopsy. Normal liver tissue samples from two males aged 69 and 78 (kindly provided by Dr. Ray Yeung, University of Washington Department of Surgery, Seattle, WA) were used as controls. Total DNA was isolated from tissue by a modified ‘salting out’ protocol: in brief, frozen liver tissue was macerated in 3 ml of lysis buffer (0.1 M Tris-HCl (pH7.5), 0.3 M NaCl, 50 mM EDTA), followed by the addition of 12.5 μl of 20 mg/ml proteinase K and 100 ul of 20% SDS and incubation overnight at 37 °C. Saturated NaCl (6 M, 1 ml) was then added to samples, followed by vigorous vortexing and centrifugation at 2000 rpm for 20 min. Supernatants were transferred to a clean 15 ml tube, to which two vol 100% ethanol was added followed by gentle end over end mixing. DNA precipitates were rinsed by transfer to a clean 5 ml tube containing 2 ml 70% ethanol, then air-dried for 3–5 min before being resolubilized in 1 ml TE buffer (10 mM Tris-HCl (pH 7.5), 1 mM EDTA) by gentle mixing overnight at room temperature. Resuspended DNA samples were analyzed on an Agilent 2200 TapeStation to determine whether DNA samples were of adequate quality for mitochondrial sequencing and telomere analysis.

### Telomere length measurements

Telomere length was measured in liver DNA samples isolated from WS patients and control donors by qPCR as previously described^25^ with minor modifications^57^. For each sample, PCR amplification was performed to amplify either telomeric DNA or the *RPLP0* large ribosomal protein gene (Gene ID: 6175) located on human chromosome 12, a single copy autosomal control against which we normalized starting DNA amounts. All samples were run in triplicate (2.5 ng of DNA in each reaction), and the median C_t_ (cycle threshold) was used for subsequent calculations. A four-point standard curve (2-fold serial dilutions, from 5 ng to 0.625 ng of DNA) was included in both PCRs to allow the transformation of C_t_ into nanograms of DNA. The amount of telomeric DNA was divided by the amount of *RPLP0* control gene DNA to generate a relative measurement of the telomere length in a sample.

### mtDNA sequence analysis

Duplex Sequencing of liver mtDNA isolated from WS patients and control donors was performed as previously described^32^,^33^ with modification. Briefly, ~100 ng of total DNA was sonicated in 60 μL of nuclease free distilled water using a Covaris AFA system with a duty cycle of 10%, intensity 5, cycles/burst 100, time 20 sec × 5 at 4 °C. After sonication, each sample was subjected to end-repair and 3′-dA-tailing using the NEBNext Ultra End-Repair/dA-Tailing Module (New England Biolabs) according to the vendor’s instructions. Duplex Sequencing adapters (2 μL of a 15 μM stock prepared as described in ref. 31) were ligated to end-repaired DNA samples using the NEBNext Ultra Ligation Module (New England Biolabs) according the vendor’s instructions. Samples were then purified to remove excess adapters using AgenCourt AmpureXP magnetic beads prior to PCR-amplification as previously described^33^. IDT xGen Lockdown probes (IDT) specific for the human mitochondrial genome were used to isolate mtDNA for 101 bp long paired-end sequencing on an Illumina HiSeq2500 (See Table S1 for probe sequences).

Sequencing reads were aligned against the human genome (hg19) using the Burrows-Wheeler Aligner and Samtools^58^ and a previously described custom processing workflow^33^. Reads not uniquely mapping to the human mtDNA genome were excluded from further analysis. After processing, we called *de novo* somatic mutations using a clonality cutoff that excluded variants occurring at a frequency of >1% at all base pair positions with a post-processing depth of ≥100. We determined the frequency of mtDNA variants by dividing the number of variants by the total number of basepairs sequenced in each sample, then scored specific mutation types only once at each mtDNA position in order to determine the spectrum of mtDNA mutations in tissue samples.

### WS patient tissue immunostaining

In order to identify and characterize senescent cells in tissue, FFPE skin samples were immunostained with a range of putative senescence markers to identify two, p16^36^,^59^ and DEC1^46^,^47^,^60^, for which we had well-characterized antisera and could demonstrate reproducible immuno-staining of WS patient tissue samples and matched control tissue of comparable vintage (autopsy tissue and blocks were originally generated between 1973–2009).

A commercially available tissue microarray (TMA SK244, Biomax, MD USA) containing normal skin from a wide range of donor ages was used as an immunostaining control. This TMA included samples from abdominal skin, as well as skin from additional sites (e.g., hand). For p16 immunostaining we used a CINtec histology kit (Ventana, AZ, USA), and for DEC1 staining a LSAB kit (Dako), both according to manufacturers’ instructions. Briefly, slides were baked, de-paraffinized, and hydrated. Antigen retrieval was performed by incubating the slides in sodium citrate in a steamer at 99–100 °C for 20 min, followed by cooling to room temperature for 20 min. After peroxidase and protein block, slides were incubated with primary antibodies for 30 min. These were a mouse monoclonal antibody clone E6H4 for p16 (provided with the CINtec histology kit, Ventana, AZ, USA), and a rabbit polyclonal anti-DEC1 antibody (a generous gift from Dr. Adrian Harris, University of Oxford). Slides were then processed using CINtec and LSAB kits for 3,3′-diaminobenzidine (DAB) chromogen substrate staining, followed by hematoxylin counter-staining, dehydration and mounting for visual cell counting and photomicrography.

## Additional Information

**Accession Codes:** Mutation data have been deposited under Submission Record ‘SUB1321610, Monnat Lab’ in ClinVar (http://www.ncbi.nlm.nih.gov/ clinvar/) on 28 January 2016, and confirmed with ClinVar on 2 Feb 2016 (https://submit.ncbi.nlm.nih.gov/subs/variation/SUB1321610/overview).

**How to cite this article**: Tokita, M. *et al*. Werner syndrome through the lens of tissue and tumour genomics. *Sci. Rep.* **6**, 32038; doi: 10.1038/srep32038 (2016).

## Supplementary Material

Supplementary Information

## Figures and Tables

**Table 1:** Werner syndrome autopsy study patient cohort.

Patient	Sex	Ancestry	*WRN* mutations*	WRN protein changes†	Cause of death (age, yrs)	Neoplasms‡
1+	F	Japanese	c.3139-1G>C c.3139-1G>C	p.G1047fs*14 p.G1047fs*14	fulminant broncho-pneumonia, pulmonary edema (57)	none
2+	F	Japanese	c.3139-1G>C c.3139-1G>C	p.G1047fs*14 p.G1047fs*14	cardiac failure secondary to pneumonia (51)	meningiomas
3	M	Japanese	c.1336C>T c.3139-1G>C	p.R369* p.G1047fs*14	pneumonia complicating metastatic pancreatic adeno-carcinoma (43)	pancreatic adenocarcinoma
4	F	English	c.1336C>T c.1336C>T	p.R369* p.R369*	metastatic pancreatic adenocarcinoma (47)	breast cancer, melanoma NOS, pancreatic adeno- carcinoma, pulmonary carcinoid

*WRN mutations identified in our four patients were determined by Dr. Junko Oshima at the International Registry of Werner Syndrome (http://www.wernersyndrome.org/) and have been deposited in ClinVar (http://www.ncbi.nlm.nih.gov/clinvar/) on 28 January 2016 under Submission Record SUB1321610, Monnat Lab.

^+^Patients 1 and 2 are full sisters.

^†^The symbol ‘*’ indicates protein termination as a result of the indicated mutation directly creating a stop codon (e.g., p.R369*) or creating a frameshift leading to a stop codon after the inclusion of 14 additional amino acids in the truncated, mutant WRN protein (p.G1047fs*14).

^‡^Neoplasms identified at the time of autopsy or previously reported in the same patient.

**Table 2:** Clinical findings in four autopsied Werner syndrome patients.

Cardinal features	Patient 1	Patient 2	Patient 3	Patient 4
cataracts	+	+	+	+
dermatologic pathology	+	+	+	+
short stature	+	+	+	+
consanguineous parents/affected sib	+	+	−	−
prematurely gray/thin scalp hair	+	+	+	+
Additional features				
diabetes mellitus	+	+	+	+
hypogonadism	+	+	NA	NA
osteoporosis	+	+	NA	+
osteosclerosis of distal phalanges	+	+	NA	NA
soft tissue calcification	+	+	NA	NA
premature atherosclerosis	+	+	+*	+#
WS-associated malignancy	−	meningioma	−	melanoma
voice changes	+	+	+	+
flat feet	NA	NA	+	+

Present (+); absent (−); not ascertained (NA).

*Mild atherosclerosis on autopsy at age 43 yrs, with a history of cerebral infarct at age 35 yrs.

^#^Mild to focally severe atherosclerotic involvement of aorta at autopsy at age 47 yrs.

**Table 3:** Pathologic findings in four autopsied Werner syndrome patients.

Histopathologic feature	Patient 1	Patient 2	Patient 3	Patient 4
Cutaneous				
atrophy of subcutaneous fat	+*	+*	+*	+*
epidermal atrophy	+	+	NA	NA
dermal fibrosis	+	+	NA	NA
hyperkeratosis	+	+	+	NA
Vascular				
atherosclerosis	+	+	+	+
arterio-/arteriolosclerosis	+	+	+	−
valvular pathology	+	+	−	−
myocardial ischemia	−	+	−	−
myocardial hypertrophy	−	−	−	−
Endocrine				
thyroid atrophy	−#	−	−	−#
thyroid adenoma	−	−	−	−
adrenal atrophy	−	−	−	−
parathyroid chief cell predomi-	−	−	NA	NA
nance				
Genitourinary				
gonadal atrophy	+	+	NA	NA
seminiferous tubule hyalinization	n/a	n/a	NA	n/a
Neurologic				
cortical atrophy	−#	−#	−	NA

Present (+); absent (−); not applicable (n/a); not ascertained (NA).

*Noted on physical exam.

^#^Small but without definite microscopic evidence of atrophy.

**Table 4:** Mutations in neoplasms from autopsied Werner syndrome patients.

Patient	Sample*	Gene/Position+	Mutation+	Reads Ref/Var+	COSMIC Listing?‡	Comments
3	pancreatic adeno-carcinoma	*KRAS* chr12:25398285	C>A/p.G12C	434/47	Yes/multiple	very common in pancreatic adenocarcinoma
		*GNAS* chr20:57415830	G>C/p.K223N§	225/24	No	validation required
		*TP53* chr17:7577141	C>T/p.G266E	794/81	Yes/multiple	
		*KIT* chr4:55594068	G>A/p.M618I‖	597/567	No	likely germline variant
4	pancreatic adeno-carcinoma	*KRAS* chr12:25398284	C>T/p.G12D	541/60	Yes/multiple	very common in pancreatic adenocarcinoma
		*SMAD4* chr18:48603032	C>T/p.R445X	441/100	Yes/multiple	also potential LOH
		*TP53* chr17:7577085	C>T/p.E285K	356/68	Yes/multiple	also potential LOH
		*GNA11* chr19:3094712	C>G/p.I21M	157/15	No	validation required

LOH = loss of heterozygosity.

*Pancreatic adenocarcinoma samples for DNA preparation were macrodissected from a liver metastasis (Patient 3) and a lymph node metastasis (Patient 4).

^+^Gene positions are with reference to the UCSC Genome Browser (GRCh37/hg19) assembly. Mutations are listed with the identified base change first, followed by the altered residue in the target protein. The mutations identified in our patients have been deposited in ClinVar (http://www.ncbi.nlm.nih.gov/clinvar/) on 28 January 2016 under Submission Record SUB1321610, Monnat Lab.

^‡^COSMIC: YES = mutation is listed in the Catalog of Somatic Mutations in Cancer database, http://cancer.sanger.ac.uk/cosmic, with ‘multiple’ indicating the presence of several COSMIC records for this mutation, though often in different tumor types.

^§^This needs to be further validated as this position is known to be artifact-prone.

^‖^KIT p.M618I variant is a rare germline variant (3 examples listed in the ExAC browser) and probably a rare benign variant.
