# Supplementary material for: Werner syndrome through the lens of tissue and tumour genomics
Source: Sci Rep. 2016 Aug 25;6:32038. doi: 10.1038/srep32038 (PMC4997333; doi:10.1038/srep32038)

Scientific Reports
revised 20160721

Supplementary Information

'Werner syndrome through the lens of tissue and tumors'

Mari Tokita, Scott R. Kennedy, Rosa Ana Risques, Stephen G. Chun, Colin Pritchard, Junko Oshima, Yan Liu, Peter K. Bryant-Greenwood, Piri Welcsh and Raymond J. Monnat, Jr.

Files:

1. **Supplementary Methods.** Detailed Werner syndrome patient clinical synopses
2. **Supplementary Results.** Detailed Werner syndrome patient autopsy findings
3. **Supplementary Table S1:** IDT xGen Lockdown™ probes to capture human mtDNA for sequencing.
4. **Supplementary Table S2:** Genes included in the UW-Oncoplex version 4 platform that are known when mutant or altered to modulate genomic stability.
5. **Supplementary Figure 1:** p16 immunostaining of control skin samples.

1. Supplementary Methods. Werner syndrome patient clinical synopses

Patient 1: Patient 1 was a woman of Japanese-American ancestry with clinically-diagnosed Werner syndrome who died at age 57 of fulminant bronchopneumonia and pulmonary edema. She has been reported as proband M8a/HMc in (Epstein et al. 1966)(UW autopsy #A-73-58), was born to non-consanguineous parents, and was the eldest in a sibship of nine that included two other sisters affected by Werner syndrome. The patient had no reported childhood medical concerns except for cessation of growth at age 13. She had menarche at age 12 and normal menses with no pregnancies up to age 44. In her early twenties, she underwent surgical

excision of bilateral cataracts followed by enucleation of the right eye for acute glaucoma. Around the same time, she noted onset of generalized increase in skin pigmentation accompanied by graying and thinning of hair. At age 31 the patient was diagnosed with diabetes mellitus that was treated with diet alone. In her late thirties she developed multiple, painful skin ulcers of the feet and progressive joint stiffness of the ankles, toes, and elbows. She developed a hoarse, high-pitched voice in her late forties.

Patient 1 on physical examination at age 48 was 142 cm tall, weighed 30.2 kg, and appeared decades older than her stated age. Her scalp hair was sparse and gray, and her skin was generally thin and hyperpigmented. Blood pressure was 170/80 and there was a 2/6 blowing early systolic murmur on cardiac exam. Her extremities were extremely thin with profound muscular atrophy, but her strength remained intact. The skin over her lower extremities was taut and smooth, and multiple cutaneous ulcers were present on both feet. She had reduced mobility of the ankle joints and superiomedial displacement of the fifth toes bilaterally. The patient had normal deep tendon reflexes, but diminished vibratory sense and two-point discrimination as well as hyperesthesia to pin-prick of the toes and feet.

Patient 1 was admitted several times in her mid-forties for complications related to lower extremity ulcers that were refractory to treatment, and ultimately underwent a right above-the-knee amputation at age 54. Two weeks prior to death at age 57 she was admitted and treated for bilateral pneumonia, dehydration, and infected decubitus ulcers. She was discharged in stable condition to her nursing home but died shortly thereafter.

Patient 2: Patient 2 was the younger sister of Patient 1 above, and was previously reported as Patient M8c/MI in (Epstein et al. 1966)(UW autopsy #A-78-82). She was diagnosed clinically with Werner syndrome, and died at age 51 from cardiac complications secondary to pulmonary bronchopneumonia.

The patient developed normally without reported problems during childhood except for short stature, generalized hyperpigmentation and dry skin. Menarche occurred at 10 years and she had irregular menses until age 32. She noted graying of hair in her late teens, followed by hair loss. In her early twenties, she developed a high-pitched voice, underwent surgical treatment for bilateral cataracts at age 25, and noted gradual tightening of the skin of the lower extremities with callus formation. Foot ulcerations developed in her early 30s along with progressive joint contractures and immobility.

Patient 2 on physical exam at age 34 weighed 26.9 kg, had sparse gray hair, and appeared decades older than her stated age with sparse gray hair and diffusely hyperpigmented skin. She had multiple palpable thyroid nodules. The skin over her distal lower extremities was thin and tight with ulcerations and calluses. Muscular atrophy of all extremities and a right knee contracture were noted. Plain film radiographs demonstrated scoliosis and osteoporosis.

She was hospitalized at age 43 for infected ulcerations and poor performance status, treated with conservative measures and discharged. Several months later she was readmitted for progressive contractures involving multiple joints, and severe ulceration of the feet that was treated definitively with bilateral above-the-knee amputations. At age 51, she was admitted for the treatment of decubitus ulcers. The patient had a third admission for infected pressure necrosis of the left elbow, which was treated surgically. Her final admission, at age 51 was to treat multiple decubitus ulcers, was complicated by a hospital-acquired pneumonia with secondary cardiac failure that led to death.

Patient 3: Patient 3 was a previously reported 43-year-old male of Japanese-American ancestry with metastatic pancreatic cancer who had biallelic loss-of-function mutations of the *WRN* gene confirmed by the Werner Syndrome International Registry (Chun et al. 2011)(Table 1). At the time of presentation the patient was cachectic, with abdominal pain, bilious emesis

and a non-productive cough. On examination, he appeared decades older than his stated age. The patient's CA-19-9 level was 1,250, which, together with a CEA level of 8.9, suggested pancreatic malignancy. A CT-scan of the abdomen demonstrated a mass in the head of the pancreas, together with likely liver metastases. A pancreatic head biopsy revealed moderately-differentiated pancreatic adenocarcinoma which expressed cytokeratin 7. Initial molecular analysis of *KRAS* by PCR amplification and sequencing and *EGFR* by fluorescent *in situ* hybridization did not reveal mutation or copy number change (Chun et al. 2011). Shortly after receiving the diagnosis of metastatic pancreatic cancer, the patient died of overwhelming pneumonia and sepsis.

Patient 4: Patient 4 was a Caucasian woman of English descent who was born to unaffected, non-consanguineous parents. She was diagnosed with molecularly-confirmed Werner syndrome at age 42, and died of metastatic pancreatic adenocarcinoma at age 47. Sequencing of the *WRN* gene identified a homozygous mutation resulting in a stop codon in place of arginine at WRN amino acid 369 (Table 1). Western blot analysis performed by the Werner Syndrome International Registry confirmed the absence of WRN protein from patient cells.

Clinical records indicate Patient 4 had no significant childhood medical concerns. She reported normal menarche at age 13, and at age 15 was diagnosed with bilateral cataracts that were surgically excised at age 30. The patient also noted thinning and graying of hair beginning in her teens. At age 34 she was diagnosed with node-positive malignant melanoma of the left ear that was treated with radical neck dissection and chemotherapy. She underwent a hysterectomy at age 37 for fibroids. At an office visit at age 40, she reported a medical history of type 2 diabetes mellitus, hypothyroidism, depression and chronic foot ailments including reduced mobility of the foot joints, multiple corrective surgeries for bilateral hammer toe and foot

pain with standing and walking. She had a history of osteoporosis with greater involvement of long bones than of vertebral bodies.

Patient 4 on physical exam at age 41 was 156.5 cm tall and weighed 52.2 kg. She had a prematurely aged appearance with graying scalp hair and thin, wasted extremities. She had ‘pinched’ facial features, tight and atrophic skin, and a hoarse voice. Apart from elevated blood pressure (160/70), her cardiovascular exam was unremarkable. At age 42 the patient was diagnosed with breast cancer and was treated with mastectomy. At age 46 she was diagnosed with metastatic pancreatic adenocarcinoma which eventually led to her demise.

2. Supplementary Results.Werner syndrome patient autopsy findings

Patient 1: On external exam Patient 1 had sparse gray scalp hair, was emaciated, and appeared decades older than her documented age. Her right eye was replaced by a prosthesis. Her skin was atrophic and sclerotic with scattered hyper- and hypopigmented patches. She had contractures of her upper and left lower extremities. Her right leg was amputated above the knee with ulceration of the stump. Several decubitus ulcers were present over the sacrum, buttocks and left foot. Her breasts were atrophic.

On internal exam all of her organs appeared small. The coronary ostia were slightly narrowed by atherosclerosis but the coronary vessels were patent. Atherosclerotic and partially calcified plaques were present on both aortic and mitral valves. The myocardium was normal. The intimal thoracic aorta demonstrated 60% involvement by calcified and partially ulcerated atherosclerotic plaques, whereas 100% of the intimal abdominal aorta and iliac arteries showed atherosclerotic involvement. Patchy, firm yellow-gray areas were present in both lungs. The thyroid was small. Parathyroid glands were of normal size. The pancreas had a normal lobular pattern with multiple tiny white spots in the parenchyma. The ovaries were fibrotic and the endometrium atrophic.

The brain was small, but displayed normal gyral and sulcal patterns without evidence of atrophy. Mild atherosclerotic change of the cerebral vessels was evident. Two soft brain lesions of unclear significance were noted in the left caudate nucleus and left cingulate gyrus.

Histopathologic exam demonstrated marked intimal and medial fibrosis of the coronary arteries and aorta, together with marked medial fibrosis of medium-sized peripheral arteries. The lungs showed chronic interstitial pneumonia and acute confluent necrotizing bronchopneumonia with pulmonary congestion and edema. Mucosa of the larynx was normal. The stomach and small intestine showed mucosal atrophy with chronic inflammation. Congestion was evident in the spleen and liver, the latter of which also had moderate fatty change. Microscopic evaluation of the thyroid, parathyroid, adrenal glands, and pancreas was normal. Arteriosclerosis and arteriolosclerosis of the kidneys was present together with marked atrophy and fibrosis of the endometrium, myometrium, and ovaries. Microscopic evaluation of the skin revealed dermal fibrosis.

Patient 2: On external exam, Patient 2 was thin and frail with atrophic and sclerotic skin, marked thinning of the nose, ears, and eyelids, and a paucity of body hair. She had patchy hyperpigmentation of the upper extremities, bilateral above-the-knee amputations, and flexion contractures of the hands and both elbows. She also had multiple ulcers over the ischia, right greater trochanter, left hand, and groin. Her breasts were underdeveloped.

On internal exam Patient 2's heart was small with one new and one remote posterior subendocardial infarction. The tricuspid, mitral, and pulmonary valves were normal, but the aortic valve was severely calcified. There was atherosclerotic disease involving the right coronary artery and aorta with plaque ulceration of the lower thoracic and abdominal aorta. Diffuse pulmonary congestion and edema were present in the lungs, and palpation revealed irregular firm areas consistent with bronchopneumonia. No gross defects of the larynx were noted. Two calcified nodules were identified in the thyroid parenchyma. The adrenal glands

were of normal size. The gastrointestinal tract including the pancreas was unremarkable. The kidneys appeared small, but had no focal lesions. Both ovaries and the uterus were small, and the vagina appeared atrophic. Neuropathologic exam showed marked cerebral atrophy and multiple meningiomas.

Histopathologic exam of the heart confirmed presence of both recent and old infarcts in the posterior septum together with abundant intra-cytoplasmic golden-brown lipochrome pigment in myocardial cells. Atherosclerotic disease was extensive, with involvement of the coronary ostia, right coronary, left main, and proximal left circumflex arteries. Microscopic exam of the lungs confirmed bilateral confluent necrotizing bronchopneumonia. Sectioning of the thyroid showed a well-circumscribed area of necrosis and small vessel disease with obliteration of luminal space. Sectioned parathyroid and adrenal tissues appeared normal. There was evidence of end-stage renal disease with arteriosclerosis and arteriolosclerosis and acute cystitis. The ovaries and uterus were markedly atrophic. Microscopy of skin sections revealed dermal thickening with fibrous tissue surrounding sweat glands.

Patient 3: A rapid autopsy was performed on Patient 3 using sterile technique and beginning 30 minutes post-mortem. Pertinent findings on external exam included generalized cachexia and a 2 cm sacral ulcer. Multiple organ systems were affected. The heart had mild calcific atherosclerosis of the major vessels, with unremarkable heart valves. Examination of the right and left lungs showed congestion and consolidation, suggestive of bilateral aspiration pneumonia. The thyroid and adrenal glands were unremarkable. A 2.2 cm mass was found in the uncinate process of the pancreas, and multiple well-circumscribed tan nodules were found in the liver. The brain showed mild non-occlusive atherosclerosis of the major cerebral blood vessels, arteriosclerosis and softening of the left parietal-occipital region with collapse of the cortical surface and hemosiderin staining, consistent with a remote infarct of the left cerebral hemisphere. Associated atrophy of the left midbrain, pons, and medulla was present.

Histopathologic analysis of the heart demonstrated focal myocyte hypertrophy of the left ventricle and interventricular septum. Mild calcific atherosclerosis was present in the aorta, bilateral coronary and left anterior descending arteries. The lungs displayed organizing pneumonia with multiple, bilateral pulmonary adenocarcinoma metastases. The pancreatic and liver masses were consistent with primary and metastatic pancreatic adenocarcinoma, respectively. Chronic pancreatitis was also present.

Patient 4: On external exam Patient 4 was cachectic and jaundiced, had wispy white scalp hair and appeared older than her 47 years of age. She had several healed surgical wounds consistent with a prior history of multiple cancer surgeries. She had marked torsion and medial displacement of all five toes on both feet.

On internal exam her heart showed moderate right ventricular dilation and focal areas of calcific atherosclerotic plaques within the left anterior descending and right coronary arteries. The aorta had moderate to focally severe calcific atherosclerotic plaques with areas of ulceration throughout its length. Her heart valves were delicate, pliable, and normally formed. The lungs had multiple, bilateral nodules involving the hilum, pleura, and parenchyma. Tracheal mucosa was reportedly normal. The left breast was surgically absent, and replaced by a prosthetic implant. The thyroid gland was small and the parathyroid glands slightly enlarged. The adrenal glands appeared normal. The esophageal and gastric walls were fibrotic. An 8 x 6 cm firm mass was found in the head of the pancreas, with extension into periaortic and periceliac arterial tissue causing extrahepatic bile duct obstruction. The liver had a cobblestoned appearance with focal areas of chalky gray-tan discoloration. The uterus, fallopian tubes, and bilateral ovaries were absent.

Histopathologic exam of the heart revealed intact myocardial architecture with no pathologic change. Microscopic analysis of nodules in both lungs demonstrated metastatic adenocarcinoma. In addition a 0.7cm carcinoid tumor was identified in the left lung upper lobe.

Microscopic exam of thyroid showed fibrosis and residual atrophic thyroid follicles. The infiltrating pancreatic mass was confirmed to be pancreatic adenocarcinoma. Extensive necrosis secondary to biliary obstruction was present in the liver, together with multifocal metastases.

Supporting Table 1: IDT xGen Lockdown probes to capture human mtDNA for sequencing

Name	*Sequence*
Hs_mtDNA_r1.1_1	/5Biosg/GATCACAGGTCTATCACCCTATTAACCACTCACGGGAGCTCTCCATGCATTTGGTATTTTCGTCTGGGGGGTATGCACGCGATAGCATTGCGAGACGCTGGAGCCGGAGCACCCTATGTC
Hs_mtDNA_r1.1_2	/5Biosg/CGCAGTATCTGTCTTTGATTCCTGCCTCATCCTATTATTTATCGCACCTACGTTCAATATTACAGGCGAACATACTTACTAAAGTGTGTTAATTAATTAATGCTTGTAGGACATAATAAT
Hs_mtDNA_r1.1_3	/5Biosg/ATAACAATTGAATGTCTGCACAGCCACTTTCCACACAGACATCATAACAAAAAATTTCCACCAAACCCCCCCTCCCCCGCTTCTGGCCACAGCACTTAAACACATCTCTGCCAAACCCCA
Hs_mtDNA_r1.1_4	/5Biosg/AAAAACAAAGAACCCTAACACCAGCCTAACCAGATTTCAAATTTTATCTTTTGGCGGTATGCACTTTTAACAGTCACCCCCCAACTAACACATTATTTTCCCCTCCCACTCCCATACTAC
Hs_mtDNA_r1.1_5	/5Biosg/ACTAATCTCATCAATACAACCCCCGCCCATCCTACCCAGCACACACACACCGCTGCTAACCCCATACCCCGAACCAACCAAACCCCAAAGACACCCCCCACAGTTTATGTAGCTTACCTC
Hs_mtDNA_r1.1_6	/5Biosg/CCTCAAAGCAATACACTGAAAATGTTTAGACGGGCTCACATCACCCCATAAACAAATAGGTTTGGTCCTAGCCTTTCTATTAGCTCTTAGTAAGATTACACATGCAAGCATCCCCGTTCC
Hs_mtDNA_r1.1_7	/5Biosg/CCAGTGAGTTCACCCTCTAAATCACCACGATCAAAAGGAACAAGCATCAAGCACGCAGCAATGCAGCTCAAAACGCTTAGCCTAGCCACACCCCCACGGGAAACAGCAGTGATTAACCTT
Hs_mtDNA_r1.1_8	/5Biosg/TTAGCAATAAACGAAAGTTTAACTAAGCTATACTAACCCCAGGGTTGGTCAATTTCGTGCCAGCCACCGCGGTCACACGATTAACCCAAGTCAATAGAAGCCGGCGTAAAGAGTGTTTTA
Hs_mtDNA_r1.1_9	/5Biosg/AGATCACCCCCTCCCCAATAAAGCTAAAACTCACCTGAGTTGTAAAAAACTCCAGTTGACACAAAATAGACTACGAAAGTGGCTTTAACATATCTGAACACACAATAGCTAAGACCCAAA
Hs_mtDNA_r1.1_10	/5Biosg/AACTGGGATTAGATACCCCACTATGCTTAGCCCTAAACCTCAACAGTTAAATCAACAAAACTGCTCGCCAGAACACTACGAGCCACAGCTTAAAACTCAAAGGACCTGGCGGTGCTTCAT
Hs_mtDNA_r1.1_11	/5Biosg/TATCCCTCTAGAGGAGCCTGTTCTGTAATCGATAAACCCCGATCAACCTCACCACCTCTTGCTCAGCCTATATACCGCCATCTTCAGCAAACCCTGATGAAGGCTACAAAGTAAGCGCAA
Hs_mtDNA_r1.1_12	/5Biosg/AAGTACCCACGTAAAGACGTTAGGTCAAGGTGTAGCCCATGAGGTGGCAAGAAATGGGCTACATTTTCTACCCCAGAAAACTACGATAGCCCTTATGAAACTTAAGGGTCGAAGGTGGAT
Hs_mtDNA_r1.1_13	/5Biosg/TTTAGCAGTAAACTAAGAGTAGAGTGCTTAGTTGAACAGGGCCCTGAAGCGCGTACACACCGCCCGTCACCCTCCTCAAGTATACTTCAAAGGACATTTAACTAAAACCCCTACGCATTT
Hs_mtDNA_r1.1_14	/5Biosg/TTATATAGAGGAGACAAGTCGTAACATGGTAAGTGTACTGGAAAGTGCACTTGGACGAACCAGAGTGTAGCTTAACACAAAGCACCCAACTTACACTTAGGAGATTTCAACTTAACTTGA
Hs_mtDNA_r1.1_15	/5Biosg/ACCGCTCTGAGCTAAACCTAGCCCCAAACCCACTCCACCTTACTACCAGACAACCTTAGCCAAACCATTTACCCAAATAAAGTATAGGCGATAGAAATTGAAACCTGGCGCAATAGATAT
Hs_mtDNA_r1.1_16	/5Biosg/TAGTACCGCAAGGGAAAGATGAAAAATTATAACCAAGCATAATATAGCAAGGACTAACCCCTATACCTTCTGCATAATGAATTAACTAGAAATAACTTTGCAAGGAGAGCCAAAGCTAAG
Hs_mtDNA_r1.1_17	/5Biosg/AGACCCCCGAAACCAGACGAGCTACCTAAGAACAGCTAAAAGAGCACACCCGTCTATGTAGCAAAATAGTGGGAAGATTTATAGGTAGAGGCGACAAACCTACCGAGCCTGGTGATAGCT
Hs_mtDNA_r1.1_18	/5Biosg/TGGTTGTCCAAGATAGAATCTTAGTTCAACTTTAAATTTGCCCACAGAACCCTCTAAATCCCCTTGTAAATTTAACTGTTAGTCCAAAGAGGAACAGCTCTTTGGACACTAGGAAAAAAC
Hs_mtDNA_r1.1_19	/5Biosg/ACCTTGTAGAGAGAGTAAAAAATTTAACACCCATAGTAGGCCTAAAAGCAGCCACCAATTAAGAAAGCGTTCAAGCTCAACACCCACTACCTAAAAAATCCCAAACATATAACTGAACTC
Hs_mtDNA_r1.1_20	/5Biosg/CCTCACACCCAATTGGACCAATCTATCACCCTATAGAAGAACTAATGTTAGTATAAGTAACATGAAAACATTCTCCTCCGCATAAGCCTGCGTCAGATTAAAACACTGAACTGACAATTA
Hs_mtDNA_r1.1_21	/5Biosg/TAACAGCCCAATATCTACAATCAACCAACAAGTCATTATTACCCTCACTGTCAACCCAACACAGGCATGCTCATAAGGAAAGGTTAAAAAAAGTAAAAGGAACTCGGCAAATCTTACCCC
Hs_mtDNA_r1.1_22	/5Biosg/CGCCTGTTTACCAAAAACATCACCTCTAGCATCACCAGTATTAGAGGCACCGCCTGCCCAGTGACACATGTTTAACGGCCGCGGTACCCTAACCGTGCAAAGGTAGCATAATCACTTGTT
Hs_mtDNA_r1.2_1	/5Biosg/ACCCTATGGAGCTTTAATTTATTAATGCAAACAGTACCTAACAAACCCACAGGTCCTAAACTACCAAACCTGCATTAAAAATTTCGGTTGGGGCGACCTCGGAGCAGAACCCAACCTCCG
Hs_mtDNA_r1.2_2	/5Biosg/GAGCAGAACCCAACCTCCGAGCAGTACATGCTAAGACTTCACCAGTCAAAGCGAACTACTATACTCAATTGATCCAATAACTTGACCAACGGAACAAGTTACCCTAGGGATAACAGCGCA
Hs_mtDNA_r1.2_3	/5Biosg/CCTAGGGATAACAGCGCAATCCTATTCTAGAGTCCATATCAACAATAGGGTTTACGACCTCGATGTTGGATCAGGACATCCCGATGGTGCAGCCGCTATTAAAGGTTCGTTTGTTCAACG
Hs_mtDNA_r1.2_4	/5Biosg/AGGTTCGTTTGTTCAACGATTAAAGTCCTACGTGATCTGAGTTCAGACCGGAGTAATCCAGGTCGGTTTCTATCTACNTTCAAATTCCTCCCTGTACGAAAGGACAAGAGAAATAAGGCC
Hs_mtDNA_r1.2_5	/5Biosg/GGACAAGAGAAATAAGGCCTACTTCACAAAGCGCCTTCCCCCGTAAATGATATCATCTCAACTTAGTATTATACCCACACCCACCCAAGAACAGGGTTTGTTAAGATGGCAGAGCCCGGT
Hs_mtDNA_r1.3_1	/5Biosg/CAATTCCTCTTCTTAACAACATACCCATGGCCAACCTCCTACTCCTCATTGTACCCATTCTAATCGCAATGGCATTCCTAATGCTTACCGAACGAAAAATTCTAGGCTATATACAACTAC
Hs_mtDNA_r1.3_2	/5Biosg/ACGCAAAGGCCCCAACGTTGTAGGCCCCTACGGGCTACTACAACCCTTCGCTGACGCCATAAAACTCTTCACCAAAGAGCCCCTAAAACCCGCCACATCTACCATCACCCTCTACATCAC
Hs_mtDNA_r1.3_3	/5Biosg/ACCGCCCCGACCTTAGCTCTCACCATCGCTCTTCTACTATGAACCCCCCTCCCCATACCCAACCCCCTGGTCAACCTCAACCTAGGCCTCCTATTTATTCTAGCCACCTCTAGCCTAGCC
Hs_mtDNA_r1.3_4	/5Biosg/CCGTTTACTCAATCCTCTGATCAGGGTGAGCATCAAACTCAAACTACGCCCTGATCGGCGCACTGCGAGCAGTAGCCCAAACAATCTCATATGAAGTCACCCTAGCCATCATTCTACTAT
Hs_mtDNA_r1.3_5	/5Biosg/TATCAACATTACTAATAAGTGGCTCCTTTAACCTCTCCACCCTTATCACAACACAAGAACACCTCTGATTACTCCTGCCATCATGACCCTTGGCCATAATATGATTTATCTCCACACTAG
Hs_mtDNA_r1.3_6	/5Biosg/AGCAGAGACCAACCGAACCCCCTTCGACCTTGCCGAAGGGGAGTCCGAACTAGTCTCAGGCTTCAACATCGAATACGCCGCAGGCCCCTTCGCCCTATTCTTCATAGCCGAATACACAAA
Hs_mtDNA_r1.3_7	/5Biosg/AACATTATTATAATAAACACCCTCACCACTACAATCTTCCTAGGAACAACATATGACGCACTCTCCCCTGAACTCTACACAACATATTTTGTCACCAAGACCCTACTTCTAACCTCCCTG
Hs_mtDNA_r1.3_8	/5Biosg/TGTTCTTATGAATTCGAACAGCATACCCCCGATTCCGCTACGACCAACTCATACACCTCCTATGAAAAAACTTCCTACCACTCACCCTAGCATTACTTATATGATATGTCTCCATACCCA
Hs_mtDNA_r1.3_9	/5Biosg/CATTACAATCTCCAGCATTCCCCCTCAAACCTAAGAAATATGTCTGATAAAAGAGTTACTTTGATAGAGTAAATAATAGGAGCTTAAACCCCCTTATTTCTAGGACTATGAGAATCGAAC
Hs_mtDNA_r1.4_1	/5Biosg/CACCTATCACACCCCATCCTAAAGTAAGGTCAGCTAAATAAGCTATCGGGCCCATACCCCGAAAATGTTGGTTATACCCTTCCCGTACTAATTAATCCCCTGGCCCAACCCGTCATCTAC
Hs_mtDNA_r1.4_2	/5Biosg/CTCTACCATCTTTGCAGGCACACTCATCACAGCGCTAAGCTCGCACTGATTTTTTACCTGAGTAGGCCTAGAAATAAACATGCTAGCTTTTATTCCAGTTCTAACCAAAAAAATAAACCC
Hs_mtDNA_r1.4_3	/5Biosg/CTCGTTCCACAGAAGCTGCCATCAAGTATTTCCTCACGCAAGCAACCGCATCCATAATCCTTCTAATAGCTATCCTCTTCAACAATATACTCTCCGGACAATGAACCATAACCAATACTA
Hs_mtDNA_r1.4_4	/5Biosg/TACCAATCAATACTCATCATTAATAATCATAATAGCTATAGCAATAAAACTAGGAATAGCCCCCTTTCACTTCTGAGTCCCAGAGGTTACCCAAGGCACCCCTCTGACATCCGGCCTGCT
Hs_mtDNA_r1.4_5	/5Biosg/TTCTTCTCACATGACAAAAACTAGCCCCCATCTCAATCATATACCAAATCTCTCCCTCACTAAACGTAAGCCTTCTCCTCACTCTCTCAATCTTATCCATCATAGCAGGCAGTTGAGGTG
Hs_mtDNA_r1.4_6	/5Biosg/GGATTAAACCAAACCCAGCTACGCAAAATCTTAGCATACTCCTCAATTACCCACATAGGATGAATAATAGCAGTTCTACCGTACAACCCTAACATAACCATTCTTAATTTAACTATTTAT
Hs_mtDNA_r1.4_7	/5Biosg/TATTATCCTAACTACTACCGCATTCCTACTACTCAACTTAAACTCCAGCACCACGACCCTACTACTATCTCGCACCTGAAACAAGCTAACATGACTAACACCCTTAATTCCATCCACCCT
Hs_mtDNA_r1.4_8	/5Biosg/CTCCTCTCCCTAGGAGGCCTGCCCCCGCTAACCGGCTTTTTGCCCAAATGGGCCATTATCGAAGAATTCACAAAAAACAATAGCCTCATCATCCCCACCATCATAGCCACCATCACCCTC
Hs_mtDNA_r1.4_9	/5Biosg/CCTTAACCTCTACTTCTACCTACGCCTAATCTACTCCACCTCAATCACACTACTCCCCATATCTAACAACGTAAAAATAAAATGACAGTTTGAACATACAAAACCCACCCCATTCCTCCC
Hs_mtDNA_r1.4_10	/5Biosg/CCACACTCATCGCCCTTACCACGCTACTCCTACCTATCTCCCCTTTTATACTAATAATCTTATAGAAATTTAGGTTAAATACAGACCAAGAGCCTTCAAAGCCCTCAGTAAGTTGCAATA
Hs_mtDNA_r1.4_11	/5Biosg/ACTTAATTTCTGTAACAGCTAAGGACTGCAAAACCCCACTCTGCATCAACTGAACGCAAATCAGCCACTTTAATTAAGCTAAGCCCTTACTAGACCAATGGGACTTAAACCCACAAACAC
Hs_mtDNA_r1.4_12	/5Biosg/ACTTAGTTAACAGCTAAGCACCCTAATCAACTGGCTTCAATCTACTTCTCCCGCCGCCGGGAAAAAAGGCGGGAGAAGCCCCGGCAGGTTTGAAGCTGCTTCTTCGAATTTGCAATTCAA
Hs_mtDNA_r1.4_13	/5Biosg/ATATGAAAATCACCTCGGAGCTGGTAAAAAGAGGCCTAACCCCTGTCTTTAGATTTACAGTCCAATGCTTCACTCAGCCATTTTACCTCACCCCCACTGATGTTCGCCGACCGTTGACTA
Hs_mtDNA_r1.4_14	/5Biosg/ATTCTCTACAAACCACAAAGACATTGGAACACTATACCTATTATTCGGCGCATGAGCTGGAGTCCTAGGCACAGCTCTAAGCCTCCTTATTCGAGCCGAGCTGGGCCAGCCAGGCAACCT
Hs_mtDNA_r1.4_15	/5Biosg/TTCTAGGTAACGACCACATCTACAACGTTATCGTCACAGCCCATGCATTTGTAATAATCTTCTTCATAGTAATACCCATCATAATCGGAGGCTTTGGCAACTGACTAGTTCCCCTAATAA
Hs_mtDNA_r1.4_16	/5Biosg/AATCGGTGCCCCCGATATGGCGTTTCCCCGCATAAACAACATAAGCTTCTGACTCTTACCTCCCTCTCTCCTACTCCTGCTCGCATCTGCTATAGTGGAGGCCGGAGCAGGAACAGGTTG
Hs_mtDNA_r1.4_17	/5Biosg/GAACAGTCTACCCTCCCTTAGCAGGGAACTACTCCCACCCTGGAGCCTCCGTAGACCTAACCATCTTCTCCTTACACCTAGCAGGTGTCTCCTCTATCTTAGGGGCCATCAATTTCATCA
Hs_mtDNA_r1.4_18	/5Biosg/ACAACAATTATCAATATAAAACCCCCTGCCATAACCCAATACCAAACGCCCCTCTTCGTCTGATCCGTCCTAATCACAGCAGTCCTACTTCTCCTATCTCTCCCAGTCCTAGCTGCTGGC
Hs_mtDNA_r1.4_19	/5Biosg/CATCACTATACTACTAACAGACCGCAACCTCAACACCACCTTCTTCGACCCCGCCGGAGGAGGAGACCCCATTCTATACCAACACCTATTCTGATTTTTCGGTCACCCTGAAGTTTATAT
Hs_mtDNA_r1.4_20	/5Biosg/ATTCTTATCCTACCAGGCTTCGGAATAATCTCCCATATTGTAACTTACTACTCCGGAAAAAAAGAACCATTTGGATACATAGGTATGGTCTGAGCTATGATATCAATTGGCTTCCTAGGG
Hs_mtDNA_r1.4_21	/5Biosg/GTTTATCGTGTGAGCACACCATATATTTACAGTAGGAATAGACGTAGACACACGAGCATATTTCACCTCCGCTACCATAATCATCGCTATCCCCACCGGCGTCAAAGTATTTAGCTGACT
Hs_mtDNA_r1.4_22	/5Biosg/TCGCCACACTCCACGGAAGCAATATGAAATGATCTGCTGCAGTGCTCTGAGCCCTAGGATTCATCTTTCTTTTCACCGTAGGTGGCCTGACTGGCATTGTATTAGCAAACTCATCACTAG
Hs_mtDNA_r1.4_23	/5Biosg/GACATCGTACTACACGACACGTACTACGTTGTAGCCCACTTCCACTATGTCCTATCAATAGGAGCTGTATTTGCCATCATAGGAGGCTTCATTCACTGATTTCCCCTATTCTCAGGCTAC
Hs_mtDNA_r1.4_24	/5Biosg/ACACCCTAGACCAAACCTACGCCAAAATCCATTTCACTATCATATTCATCGGCGTAAATCTAACTTTCTTCCCACAACACTTTCTCGGCCTATCCGGAATGCCCCGACGTTACTCGGACT
Hs_mtDNA_r1.4_25	/5Biosg/TACCCCGATGCATACACCACATGAAACATCCTATCATCTGTAGGCTCATTCATTTCTCTAACAGCAGTAATATTAATAATTTTCATGATTTGAGAAGCCTTCGCTTCGAAGCGAAAAGTC
Hs_mtDNA_r1.4_26	/5Biosg/CCTAATAGTAGAAGAACCCTCCATAAACCTGGAGTGACTATATGGATGCCCCCCACCCTACCACACATTCGAAGAACCCGTATACATAAAATCTAGACAAAAAAGGAAGGAATCGAACCC
Hs_mtDNA_r1.5_1	/5Biosg/CATGGCCTCCATGACTTTTTCAAAAAGGTATTAGAAAAACCATTTCATAACTTTGTCAAAGTTAAATTATAGGCTAAATCCTATATATCTTAATGGCACATGCAGCGCAAGTAGGTCTAC
Hs_mtDNA_r1.5_2	/5Biosg/AAGACGCTACTTCCCCTATCATAGAAGAGCTTATCACCTTTCATGATCACGCCCTCATAATCATTTTCCTTATCTGCTTCCTAGTCCTGTATGCCCTTTTCCTAACACTCACAACAAAAC
Hs_mtDNA_r1.5_3	/5Biosg/CTAACTAATACTAACATCTCAGACGCTCAGGAAATAGAAACCGTCTGAACTATCCTGCCCGCCATCATCCTAGTCCTCATCGCCCTCCCATCCCTACGCATCCTTTACATAACAGACGAG
Hs_mtDNA_r1.5_4	/5Biosg/GTCAACGATCCCTCCCTTACCATCAAATCAATTGGCCACCAATGGTACTGAACCTACGAGTACACCGACTACGGCGGACTAATCTTCAACTCCTACATACTTCCCCCATTATTCCTAGAA
Hs_mtDNA_r1.5_5	/5Biosg/ACCAGGCGACCTGCGACTCCTTGACGTTGACAATCGAGTAGTACTCCCGATTGAAGCCCCCATTCGTATAATAATTACATCACAAGACGTCTTGCACTCATGAGCTGTCCCCACATTAGG
Hs_mtDNA_r1.5_6	/5Biosg/CTTAAAAACAGATGCAATTCCCGGACGTCTAAACCAAACCACTTTCACCGCTACACGACCGGGGGTATACTACGGTCAATGCTCTGAAATCTGTGGAGCAAACCACAGTTTCATGCCCAT
Hs_mtDNA_r1.5_7	/5Biosg/CGTCCTAGAATTAATTCCCCTAAAAATCTTTGAAATAGGGCCCGTATTTACCCTATAGCACCCCCTCTACCCCCTCTAGAGCCCACTGTAAAGCTAACTTAGCATTAACCTTTTAAGTTA
Hs_mtDNA_r1.5_8	/5Biosg/AAAGATTAAGAGAACCAACACCTCTTTACAGTGAAATGCCCCAACTAAATACTACCGTATGGCCCACCATAATTACCCCCATACTCCTTACACTATTCCTCATCACCCAACTAAAAATAT
Hs_mtDNA_r1.5_9	/5Biosg/TAAACACAAACTACCACCTACCTCCCTCACCAAAGCCCATAAAAATAAAAAATTATAACAAACCCTGAGAACCAAAATGAACGAAAATCTGTTCGCTTCATTCATTGCCCCCACAATCCT
Hs_mtDNA_r1.5_10	/5Biosg/AGGCCTACCCGCCGCAGTACTGATCATTCTATTTCCCCCTCTATTGATCCCCACCTCCAAATATCTCATCAACAACCGACTAATCACCACCCAACAATGACTAATCAAACTAACCTCAAA
Hs_mtDNA_r1.5_11	/5Biosg/AACAAATGATAACCATACACAACACTAAAGGACGAACCTGATCTCTTATACTAGTATCCTTAATCATTTTTATTGCCACAACTAACCTCCTCGGACTCCTGCCTCACTCATTTACACCAA
Hs_mtDNA_r1.5_12	/5Biosg/CCACCCAACTATCTATAAACCTAGCCATGGCCATCCCCTTATGAGCGGGCACAGTGATTATAGGCTTTCGCTCTAAGATTAAAAATGCCCTAGCCCACTTCTTACCACAAGGCACACCTA
Hs_mtDNA_r1.5_13	/5Biosg/ACACCCCTTATCCCCATACTAGTTATTATCGAAACCATCAGCCTACTCATTCAACCAATAGCCCTGGCCGTACGCCTAACCGCTAACATTACTGCAGGCCACCTACTCATGCACCTAATT
Hs_mtDNA_r1.5_14	/5Biosg/GGAAGCGCCACCCTAGCAATATCAACCATTAACCTTCCCTCTACACTTATCATCTTCACAATTCTAATTCTACTGACTATCCTAGAAATCGCTGTCGCCTTAATCCAAGCCTACGTTTTC
Hs_mtDNA_r1.5_15	/5Biosg/ACACTTCTAGTAAGCCTCTACCTGCACGACAACACATAATGACCCACCAATCACATGCCTATCATATAGTAAAACCCAGCCCATGACCCCTAACAGGGGCCCTCTCAGCCCTCCTAATGA
Hs_mtDNA_r1.5_16	/5Biosg/ACCTCCGGCCTAGCCATGTGATTTCACTTCCACTCCATAACGCTCCTCATACTAGGCCTACTAACCAACACACTAACCATATACCAATGATGGCGCGATGTAACACGAGAAAGCACATAC
Hs_mtDNA_r1.5_17	/5Biosg/CAAGGCCACCACACACCACCTGTCCAAAAAGGCCTTCGATACGGGATAATCCTATTTATTACCTCAGAAGTTTTTTTCTTCGCAGGATTTTTCTGAGCCTTTTACCACTCCAGCCTAGCC
Hs_mtDNA_r1.5_18	/5Biosg/CCCTACCCCCCAATTAGGAGGGCACTGGCCCCCAACAGGCATCACCCCGCTAAATCCCCTAGAAGTCCCACTCCTAAACACATCCGTATTACTCGCATCAGGAGTATCAATCACCTGAGC

Hs_mtDNA_r1.5_19	/5Biosg/TCACCATAGTCTAATAGAAAACAACCGAAACCAAATAATTCAAGCACTGCTTATTACAATTTTACTGGGTCTCTATTTTACCCTCCTACAAGCCTCAGAGTACTTCGAGTCTCCCTTCAC
Hs_mtDNA_r1.5_20	/5Biosg/CATTTCCGACGGCATCTACGGCTCAACATTTTTTGTAGCCACAGGCTTCCACGGACTTCACGTCATTATTGGCTCAACTTTCCTCACTATCTGCTTCATCCGCCAACTAATATTTCACTT
Hs_mtDNA_r1.5_21	/5Biosg/TTACATCCAAACATCACTTTGGCTTCGAAGCCGCCGCCTGATACTGGCATTTTGTAGATGTGGTTTGACTATTTCTGTATGTCTCCATCTATTGATGAGGGTCTTACTCTTTTAGTATAA
Hs_mtDNA_r1.5_22	/5Biosg/ATAGTACCGTTAACTTCCAATTAACTAGTTTTGACAACATTCAAAAAAGAGTAATAAACTTCGCCTTAATTTTAATAATCAACACCCTCCTAGCCTTACTACTAATAATTATTACATTTT
Hs_mtDNA_r1.5_23	/5Biosg/TGACTACCACAACTCAACGGCTACATAGAAAAATCCACCCCTTACGAGTGCGGCTTCGACCCTATATCCCCCGCCCGCGTCCCTTTCTCCATAAAATTCTTCTTAGTAGCTATTACCTTC
Hs_mtDNA_r1.5_24	/5Biosg/TTATTATTTGATCTAGAAATTGCCCTCCTTTTACCCCTACCATGAGCCCTACAAACAACTAACCTGCCACTAATAGTTATGTCATCCCTCTTATTAATCATCATCCTAGCCCTAAGTCTG
Hs_mtDNA_r1.5_25	/5Biosg/GCCTATGAGTGACTACAAAAAGGATTAGACTGAACCGAATTGGTATATAGTTTAAACAAAACGAATGATTTCGACTCATTAAATTATGATAATCATATTTACCAAATGCCCCTCATTTAC
Hs_mtDNA_r1.5_26	/5Biosg/CATAAATATTATACTAGCATTTACCATCTCACTTCTAGGAATACTAGTATATCGCTCACACCTCATATCCTCCCTACTATGCCTAGAAGGAATAATACTATCGCTGTTCATTATAGCTAC
Hs_mtDNA_r1.5_27	/5Biosg/TCTCATAACCCTCAACACCCACTCCCTCTTAGCCAATATTGTGCCTATTGCCATACTAGTCTTTGCCGCCTGCGAAGCAGCGGTGGGCCTAGCCCTACTAGTCTCAATCTCCAACACATA
Hs_mtDNA_r1.5_28	/5Biosg/TGGCCTAGACTACGTACATAACCTAAACCTACTCCAATGCTAAAACTAATCGTCCCAACAATTATATTACTACCACTGACATGACTTTCCAAAAAACACATAATTTGAATCAACACAACC
Hs_mtDNA_r1.5_29	/5Biosg/CACCCACAGCCTAATTATTAGCATCATCCCTCTACTATTTTTTAACCAAATCAACAACAACCTATTTAGCTGTTCCCCAACCTTTTCCTCCGACCCCCTAACAACCCCCCTCCTAATACT
Hs_mtDNA_r1.5_30	/5Biosg/AACTACCTGACTCCTACCCCTCACAATCATGGCAAGCCAACGCCACTTATCCAGTGAACCACTATCACGAAAAAAACTCTACCTCTCTATACTAATCTCCCTACAAATCTCCTTAATTAT
Hs_mtDNA_r1.5_31	/5Biosg/TAACATTCACAGCCACAGAACTAATCATATTTTATATCTTCTTCGAAACCACACTTATCCCCACCTTGGCTATCATCACCCGATGAGGCAACCAGCCAGAACGCCTGAACGCAGGCACAT
Hs_mtDNA_r1.5_32	/5Biosg/ACTTCCTATTCTACACCCTAGTAGGCTCCCTTCCCCTACTCATCGCACTAATTTACACTCACAACACCCTAGGCTCACTAAACATTCTACTACTCACTCTCACTGCCCAAGAACTATCAA
Hs_mtDNA_r1.5_33	/5Biosg/ACTCCTGAGCCAACAACTTAATATGACTAGCTTACACAATAGCTTTTATAGTAAAGATACCTCTTTACGGACTCCACTTATGACTCCCTAAAGCCCATGTCGAAGCCCCCATCGCTGGGT
Hs_mtDNA_r1.5_34	/5Biosg/TCAATAGTACTTGCCGCAGTACTCTTAAAACTAGGCGGCTATGGTATAATACGCCTCACACTCATTCTCAACCCCCTGACAAAACACATAGCCTACCCCTTCCTTGTACTATCCCTATGA
Hs_mtDNA_r1.5_35	/5Biosg/GGCATAATTATAACAAGCTCCATCTGCCTACGACAAACAGACCTAAAATCGCTCATTGCATACTCTTCAATCAGCCACATAGCCCTCGTAGTAACAGCCATTCTCATCCAAACCCCCTGA
Hs_mtDNA_r1.5_36	/5Biosg/AAGCTTCACCGGCGCAGTCATTCTCATAATCGCCCACGGGCTTACATCCTCATTACTATTCTGCCTAGCAAACTCAAACTACGAACGCACTCACAGTCGCATCATAATCCTCTCTCAAGG
Hs_mtDNA_r1.5_37	/5Biosg/ACTTCAAACTCTACTCCCACTAATAGCTTTTTGATGACTTCTAGCAAGCCTCGCTAACCTCGCCTTACCCCCCACTATTAACCTACTGGGAGAACTCTCTGTGCTAGTAACCACGTTCTC
Hs_mtDNA_r1.5_38	/5Biosg/CTGATCAAATATCACTCTCCTACTTACAGGACTCAACATACTAGTCACAGCCCTATACTCCCTCTACATATTTACCACAACACAATGGGGCTCACTCACCCACCACATTAACAACATAAA
Hs_mtDNA_r1.5_39	/5Biosg/AACCCTCATTCACACGAGAAAACACCCTCATGTTCATACACCTATCCCCCATTCTCCTCCTATCCCTCAACCCCGACATCATTACCGGGTTTTCCTCTTGTAAATATAGTTTAACCAAAA
Hs_mtDNA_r1.5_40	/5Biosg/CATCAGATTGTGAATCTGACAACAGAGGCTTACGACCCCTTATTTACCGAGAAAGCTCACAAGAACTGCTAACTCATGCCCCCATGTCTAACAACATGGCTTTCTCAACTTTTAAAGGAT
Hs_mtDNA_r1.5_41	/5Biosg/TAACAGCTATCCATTGGTCTTAGGCCCCAAAAATTTTGGTGCAACTCCAAATAAAAGTAATAACCATGCACACTACTATAACCACCCTAACCCTGACTTCCCTAATTCCCCCCATCCTTA
Hs_mtDNA_r1.5_42	/5Biosg/CCACCCTCGTTAACCCTAACAAAAAAAACTCATACCCCCATTATGTAAAATCCATTGTCGCATCCACCTTTATTATCAGTCTCTTCCCCACAACAATATTCATGTGCCTAGACCAAGAAG
Hs_mtDNA_r1.5_43	/5Biosg/TTATTATCTCGAACTGACACTGAGCCACAACCCAAACAACCCAGCTCTCCCTAAGCTTCAAACTAGACTACTTCTCCATAATATTCATCCCTGTAGCATTGTTCGTTACATGGTCCATCA
Hs_mtDNA_r1.5_44	/5Biosg/ATAGAATTCTCACTGTGATATATAAACTCAGACCCAAACATTAATCAGTTCTTCAAATATCTACTCATCTTCCTAATTACCATACTAATCTTAGTTACCGCTAACAACCTATTCCAACTG
Hs_mtDNA_r1.5_45	/5Biosg/TTCATCGGCTGAGAGGGCGTAGGAATTATATCCTTCTTGCTCATCAGTTGATGATACGCCCGAGCAGATGCCAACACAGCAGCCATTCAAGCAATCCTATACAACCGTATCGGCGATATC
Hs_mtDNA_r1.5_46	/5Biosg/GGTTTCATCCTCGCCTTAGCATGATTTATCCTACACTCCAACTCATGAGACCCACAACAAATAGCCCTTCTAAACGCTAATCCAAGCCTCACCCCACTACTAGGCCTCCTCCTAGCAGCA
Hs_mtDNA_r1.5_47	/5Biosg/AGCAGGCAAATCAGCCCAATTAGGTCTCCACCCCTGACTCCCCTCAGCCATAGAAGGCCCCACCCCAGTCTCAGCCCTACTCCACTCAAGCACTATAGTTGTAGCAGGAATCTTCTTACT
Hs_mtDNA_r1.5_48	/5Biosg/CATCCGCTTCCACCCCCTAGCAGAAAATAGCCCACTAATCCAAACTCTAACACTATGCTTAGGCGCTATCACCACTCTGTTCGCAGCAGTCTGCGCCCTTACACAAAATGACATCAAAAA
Hs_mtDNA_r1.5_49	/5Biosg/AAATCGTAGCCTTCTCCACTTCAAGTCAACTAGGACTCATAATAGTTACAATCGGCATCAACCAACCACACCTAGCATTCCTGCACATCTGTACCCACGCCTTCTTCAAAGCCATACTAT
Hs_mtDNA_r1.5_50	/5Biosg/TTATGTGCTCCGGGTCCATCATCCACAACCTTAACAATGAACAAGATATTCGAAAAATAGGAGGACTACTCAAAACCATACCTCTCACTTCAACCTCCCTCACCATTGGCAGCCTAGCAT
Hs_mtDNA_r1.5_51	/5Biosg/TAGCAGGAATACCTTTCCTCACAGGTTTCTACTCCAAAGACCACATCATCGAAACCGCAAACATATCATACACAAACGCCTGAGCCCTATCTATTACTCTCATCGCTACCTCCCTGACAA
Hs_mtDNA_r1.5_52	/5Biosg/AGCGCCTATAGCACTCGAATAATTCTTCTCACCCTAACAGGTCAACCTCGCTTCCCCACCCTTACTAACATTAACGAAAATAACCCCACCCTACTAAACCCCATTAAACGCCTGGCAGCC
Hs_mtDNA_r1.5_53	/5Biosg/GGAAGCCTATTCGCAGGATTTCTCATTACTAACAACATTTCCCCCGCATCCCCCTTCCAAACAACAATCCCCCTCTACCTAAAACTCACAGCCCTCGCTGTCACTTTCCTAGGACTTCTA
Hs_mtDNA_r1.5_54	/5Biosg/AACAGCCCTAGACCTCAACTACCTAACCAACAAACTTAAAATAAAATCCCCACTATGCACATTTTATTTCTCCAACATACTCGGATTCTACCCTAGCATCACACACCGCACAATCCCCTA
Hs_mtDNA_r1.5_55	/5Biosg/TCTAGGCCTTCTTACGAGCCAAAACCTGCCCCTACTCCTCCTAGACCTAACCTGACTAGAAAAGCTATTACCTAAAACAATTTCACAGCACCAAATCTCCACCTCCATCATCACCTCAAC
Hs_mtDNA_r1.5_56	/5Biosg/CCAAAAAGGCATAATTAAACTTTACTTCCTCTCTTTCTTCTTCCCACTCATCCTAACCCTACTCCTAATCACATAACCTATTCCCCCGAGCAATCTCAATTACAATATATACACCAACAA
Hs_mtDNA_r1.5_57	/5Biosg/AACAATGTTCAACCAGTAACTACTACTAATCAACGCCCATAATCATACAAAGCCCCCGCACCAATAGGATCCTCCCGAATCAACCCTGACCCCTCTCCTTCATAAATTATTCAGCTTCCT
Hs_mtDNA_r1.5_58	/5Biosg/ACACTATTAAAGTTTACCACAACCACCACCCCATCATACTCTTTCACCCACAGCACCAATCCTACCTCCATCGCTAACCCCACTAAAACACTCACCAAGACCTCAACCCCTGACCCCCAT
Hs_mtDNA_r1.5_59	/5Biosg/TGCCTCAGGATACTCCTCAATAGCCATCGCTGTAGTATATCCAAAGACAACCATCATTCCCCCTAAATAAATTAAAAAAACTATTAAACCCATATAACCTCCCCCAAAATTCAGAATAAT
Hs_mtDNA_r1.5_60	/5Biosg/AACACACCCGACCACACCGCTAACAATCAATACTAAACCCCCATAAATAGGAGAAGGCTTAGAAGAAAACCCCACAAACCCCATTACTAAACCCACACTCAACAGAAACAAAGCATACAT
Hs_mtDNA_r1.5_61	/5Biosg/CATTATTCTCGCACGGACTACAACCACGACCAATGATATGAAAAACCATCGTTGTATTTCAACTACAAGAACACCAATGACCCCAATACGCAAAACTAACCCCCTAATAAAATTAATTAA
Hs_mtDNA_r1.5_62	/5Biosg/ACCACTCATTCATCGACCTCCCCACCCCATCCAACATCTCCGCATGATGAAACTTCGGCTCACTCCTTGGCGCCTGCCTGATCCTCCAAATCACCACAGGACTATTCCTAGCCATGCACT
Hs_mtDNA_r1.5_63	/5Biosg/ACTCACCAGACGCCTCAACCGCCTTTTCATCAATCGCCCACATCACTCGAGACGTAAATTATGGCTGAATCATCCGCTACCTTCACGCCAATGGCGCCTCAATATTCTTTATCTGCCTCT
Hs_mtDNA_r1.5_64	/5Biosg/TCCTACACATCGGGCGAGGCCTATATTACGGATCATTTCTCTACTCAGAAACCTGAAACATCGGCATTATCCTCCTGCTTGCAACTATAGCAACAGCCTTCATAGGCTATGTCCTCCCGT
Hs_mtDNA_r1.5_65	/5Biosg/TGAGGCCAAATATCATTCTGAGGGGCCACAGTAATTACAAACTTACTATCCGCCATCCCATACATTGGGACAGACCTAGTTCAATGAATCTGAGGAGGCTACTCAGTAGACAGTCCCACC
Hs_mtDNA_r1.5_66	/5Biosg/CTCACACGATTCTTTACCTTTCACTTCATCTTGCCCTTCATTATTGCAGCCCTAGCAACACTCCACCTCCTATTCTTGCACGAAACGGGATCAAACAACCCCCTAGGAATCACCTCCCAT
Hs_mtDNA_r1.5_67	/5Biosg/TTCCGATAAAATCACCTTCCACCCTTACTACACAATCAAAGACGCCCTCGGCTTACTTCTCTTCCTTCTCTCCTTAATGACATTAACACTATTCTCACCAGACCTCCTAGGCGACCCAGA
Hs_mtDNA_r1.5_68	/5Biosg/CAATTATACCCTAGCCAACCCCTTAAACACCCCTCCCCACATCAAGCCCGAATGATATTTCCTATTCGCCTACACAATTCTCCGATCCGTCCCTAACAAACTAGGAGGCGTCCTTGCCCT
Hs_mtDNA_r1.5_69	/5Biosg/ATTACTATCCATCCTCATCCTAGCAATAATCCCCATCCTCCATATATCCAAACAACAAAGCATAATATTTCGCCCACTAAGCCAATCACTTTATTGACTCCTAGCCGCAGACCTCCTCAT
Hs_mtDNA_r1.5_70	/5Biosg/TTCTAACCTGAATCGGAGGACAACCAGTAAGCTACCCTTTTACCATCATTGGACAAGTAGCATCCGTACTATACTTCACAACAATCCTAATCCTAATACCAACTATCTCCCTAATTGAAA
Hs_mtDNA_r1.5_71	/5Biosg/ACAAAATACTCAAATGGGCCTGTCCTTGTAGTATAAACTAATACACCAGTCTTGTAAACCGGAGATGAAAACCTTTTTCCAAGGACAAATCAGAGAAAAAGTCTTTAACTCCACCATTAG
Hs_mtDNA_r1.5_72	/5Biosg/GCACCCAAAGCTAAGATTCTAATTTAAACTATTCTCTGTTCTTTCATGGGGAAGCAGATTTGGGTACCACCCAAGTATTGACTCACCCATCAACAACCGCTATGTATTTCGTACATTACT
Hs_mtDNA_r1.5_73	/5Biosg/GCCAGCCACCATGAATATTGTACGGTACCATAAATACTTGACCACCTGTAGTACATAAAAACCCAATCCACATCAAAACCCCCTCCCCATGCTTACAAGCAAGTACAGCAATCAACCCTC
Hs_mtDNA_r1.6_1	/5Biosg/TATCACACATCAACTGCAACTCCAAAGCCACCCCTCACCCACTAGGATACCAACAAACCTACCCACCCTTAACAGTACATAGTACATAAAGCCATTTACCGTACATAGCACATTACAGTC
Hs_mtDNA_r1.6_2	/5Biosg/ATTACAGTCAAATCCCTTCTCGTCCCCATGGATGACCCCCCTCAGATAGGGGTCCCTTGACCACCATCCTCCGTGAAATCAATATCCCGCACAAGAGTGCTACTCTCCTCGCTCCGGGCC
Hs_mtDNA_r1.6_3	/5Biosg/GCTCCGGGCCCATAACACTTGGGGGTAGCTAAAGTGAACTGTATCCGACATCTGGTTCCTACTTCAGGGTCATAAAGCCTAAATAGCCCACACGTTCCCCTTAAATAAGACATCACGATG

Supplementary Table S2: Genes included in UW-Oncoplex version 4 that when mutant or altered modulate genomic stability*.

ATM	*MDM2/4*	*TET2*
	MRE11A	*TP53*
BAP1	*MSH2/6*	
BARD1	*MTHFR*	*VHL*
BRCA1	*MUTYH*	
BRCA2	*MYC*	*WT1*
BRIP1	*MYCN*	
	MYCL1	
CCND1		
CCNE1	*NPM1*	
CDK4/6/8	*NRAS*	
CHD1		
CHEK1/2	*PALB2*	
	PML	
ERCC2/XPD	*PMS2*	
EZH2	*POLD1*	
	POLE	
FBXW7	*PTEN*	
HRAS	*RAD51C*	
	RAD51D	
IDH1/2	*RB1*	
KDM6A/UTX		
KRAS		

* The roles of specific genes included in the UW-Oncoplex panel in DNA repair, the DNA damage response and genome stability assurance are listed in Pearl *et al.*, 2015.

Tokita et al. Supplementary Figure 1 – p16 immunostaining of control skin samples.

Panel A: p16 immunostaining of normal skin sample from a 4 month old. Note the virtual lack of any detectable p16 immnostaining (this result was consistent between six different 4 month old skin samples). **Panel B:** p16 immunostaining in a normal skin sample from a 49 year old. Note weak p16 staining in this control sample, restricted to the epidermis. This staining pattern was also observed in another 49 year old control skin sample.

A B

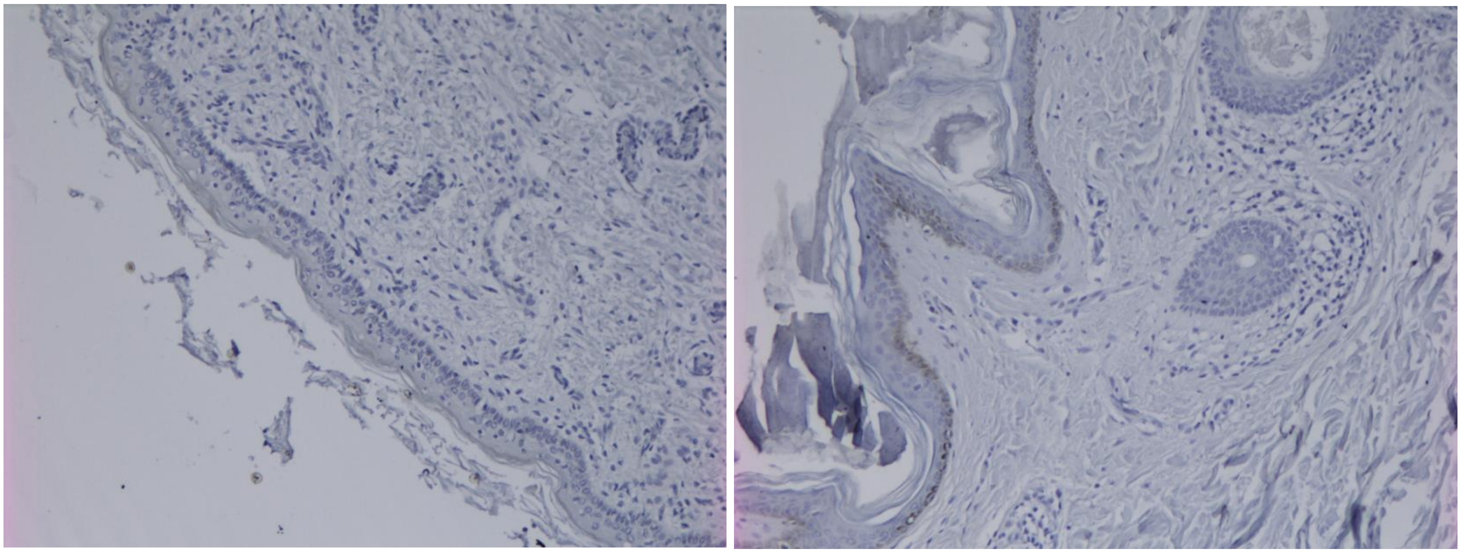

Supplement: Supplementary Information [file srep32038-s1.pdf]
